# Synthetic polymer nanocarrier enhances dsRNA stability but not RNAi efficacy in *Brassicogethes aeneus*

**DOI:** 10.1002/ps.70516

**Published:** 2026-01-19

**Authors:** Triin Kallavus, Liina Soonvald, Iris Callewaert, Ana Isabel Silva, Ericmar Ávila Dos Santos, Silva Vilumets, Riina Kaasik, Sven Vereecken, Lenny Van Daele, Peter Dubruel, Jonathan Willow, Clauvis Nji Tizi Taning, Kristof De Schutter, Eve Veromann

**Affiliations:** ^1^ Chair of Plant Health, Institute of Agricultural and Environmental Sciences, Estonian University of Life Sciences Tartu Estonia; ^2^ Department of Plants and Crops, Faculty of Bioscience Engineering Ghent University Ghent Belgium; ^3^ Center for Magnetic Resonance Research, Department of Radiology University of Minnesota Minneapolis MN USA; ^4^ Polymer Chemistry and Biomaterials Group, Department of Organic and Macromolecular chemistry Ghent University Ghent Belgium; ^5^ Pheasants Forever and Quail Forever St. James MN USA

**Keywords:** insect pest management, *Meligethes*, nanoparticles, nanotechnology, oilseed rape, RNA interference

## Abstract

**BACKGROUND:**

The use of double‐stranded RNA (dsRNA) for pest insect management presents a promising strategy for protecting crop yields, preserving ecosystem integrity and improving food security and safety. However, to address the natural instability of dsRNA, nanocarriers have been developed to enhance both its stability and cellular uptake *in vivo*.

This study evaluates the laboratory efficacy of naked *vs* complexed dsRNA in the RNAi‐mediated control of the pollen beetle *Brassicogethes aeneus*, a major insect pest of rapeseed crops. After selecting a synthetic cationic polymer that successfully protects dsRNA from degradation by *B*. *aeneus* gut nucleases, pollen beetles were orally exposed to either complexed or naked dsRNA targeting essential genes.

**RESULTS:**

Our results demonstrate that the low‐molar‐mass synthetic polymer nanocarrier PAEMA enhances the stability of dsRNA in the insect gut environment and facilitates its release. However, it does not lead to an increased mortality rate of *B. aeneus*.

**CONCLUSION:**

These findings suggest that nanocarrier systems achieving successful dsRNA complexation and enhanced stability do not necessarily result in a significant reduction of survival in pollen beetles. They also highlight the need for a deeper understanding of the complexation and release conditions specific to each dsRNA–nanocarrier system within a relevant biological context to develop effective dsRNA delivery strategies for *B. aeneus* control. © 2026 The Author(s). *Pest Management Science* published by John Wiley & Sons Ltd on behalf of Society of Chemical Industry.

## Graphical abstract

Experimental setup for dsRNA feeding in Brassicogethes aeneus to evaluate the effect of a synthetic polymer nanocarrier on RNAi efficiency. The experiment assessed whether the nanocarrier enhances RNAi after oral dsRNA delivery. (a) Stability of dsRNA–nanocarrier complexes was tested in simulated gut conditions. (b) Gene expression of αCOP and dre4 was measured in beetles fed naked *versus* nanocarrier‐bound dsRNA.

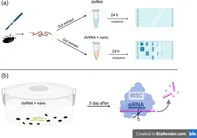

## 1 INTRODUCTION

Oilseed rape (*Brassica napus* L.) is a highly versatile crop which has gained significant importance for its applications in food (as canola/rapeseed oil), feed and biodiesel production. It is widely cultivated across North America, Europe, Asia and Australia, with its expansion driven by the global demand for biofuels.^1^, ^2^, ^3^ In 2024, the cultivation area of rapeseed in Europe approached 6 million ha, whereas in Canada it exceeded 13 million ha.^4^, ^5^, ^6^ Like many crops, rapeseed is susceptible to insect pests that can reduce its yield by ≤15%.^7^, ^8^ Among these, the pollen beetle *Brassicogethes aeneus* Fabricius stands out as a major insect pest, not only for rapeseed, but also for other cruciferous crops.^9^ During winter, *B. aeneus* adults overwinter in leaf litter, hedgerows or grassy areas.^10^, ^11^, ^12^ In early spring, they emerge and migrate to flowering plants, including rapeseed, where they feed on pollen and nectar to replenish energy and initiate egg production. The adult beetles are generalist feeders, feeding on a wide variety of wild plants from different families, yet they exhibit strong preference for brassicas for oviposition.^13^, ^14^ Female beetles lay their eggs inside flower buds of cruciferous plants, chewing a hole at the base of the bud for egg deposition.^15^ After hatching, the 1^st^‐instar larvae feed on the pollen within these buds, whereas the 2^nd^‐instar larvae consume pollen from open flowers before descending to the soil to pupate.^12^, ^13^ The most significant damage caused by *B. aeneus* occurs when adults feed on rapeseed during the green bud stage of plants, severely impacting plant development and yield.^16^ The management of *B. aeneus* has traditionally relied on synthetic pyrethroid insecticides.^16^, ^17^, ^18^, ^19^, ^20^, ^21^ However, their extensive use has led to the emergence of resistance across Europe.^22^, ^23^, ^24^, ^25^ In addition, the negative impact of pyrethroids on nontarget organisms highlights the urgent need for alternative insect pest management solutions.^26^, ^27^

RNA interference (RNAi) technology is increasingly recognized as a highly specific and ecologically sustainable tool within integrated pest management (IPM). Owing to its nucleotide sequence‐specific mode‐of‐action (MoA), RNAi can selectively target pest species without harming economically beneficial insects, a key principle in IPM.^28^, ^29^, ^30^ The potential of RNAi‐based crop protection against insect pests is demonstrated by products entering the market, such as a transgenic maize line expressing double‐stranded RNA (dsRNA) targeting western corn rootworm (*Diabrotica virgifera vergifera* LeConte)^31^ and a sprayable dsRNA biopesticide targeting Colorado potato beetle (*Leptinotarsa decemlineata* Say).^32^ Furthermore, recent advancements in the synthesis of dsRNA (cell‐free systems) have significantly enhanced scalability and reduced synthesis costs, making spray‐induced gene silencing (SIGS) a potentially cost‐effective strategy.^30^

Effective use of RNA‐based pest control products requires addressing certain technical challenges. dsRNA is vulnerable to rapid enzymatic degradation and pH fluctuations within the insect digestive tract, which can hinder effective mRNA downregulation.^33^, ^34^, ^35^, ^36^, ^37^ Owing to their size and negative charge, siRNA and dsRNA cannot be readily internalized by cells through diffusion.^38^ Instead, cellular uptake of dsRNA typically involves SID‐1–like transmembrane channels and endocytosis, including clathrin‐ and scavenger receptor–mediated pathways.^39^ However, the extent to which each of these uptake routes contributes to dsRNA uptake in insects varies greatly in different orders.^40^ To overcome these barriers, nanoparticle‐based delivery systems have been explored to stabilize, protect and efficiently deliver dsRNA.^41^ Studies have reported enhanced dsRNA delivery in insects, using various nanoparticles such as chitosan,^39^, ^40^ graphene oxide,^42^ liposomes,^43^, ^44^ clay,^45^ protein^46^ and polymers.^47^ Polymer‐based carriers, whether naturally occurring or synthetically designed, can be customized in terms of size and structure to suit specific pests and environmental conditions.^32^, ^45^, ^46^, ^47^ For example, incorporating strong basic side groups, such as guanidine, into the polymer structure improves dsRNA delivery by providing strong electrostatic interactions with the negatively charged phosphate backbone of dsRNA. This enhances stability and protects the dsRNA from degradation by nucleases.^48^, ^49^ Such approaches have been successfully tested on *Spodoptera exigua* Hübner, which has an alkaline gut environment, as demonstrated by Christiaens *et al*.^50^ In their study, the mortality of *S. exigua* increased when synthetic polymers with guanidine groups were used.

The potential of RNAi‐based control of *B*. *aeneus* has been studied by Willow *et al*.^51^, ^52^, ^53^, ^54^, ^55^ and Knorr *et al*.^56^, ^57^ Collectively, these studies tested various dsRNAs on both adult and larval stages of *B*. *aeneus*, and through various methods of dietary exposure. Notably, RNAi efficacy was examined under semi‐natural conditions by exposing beetles to plants sprayed with dsRNA.^58^

The aim of this study was to compare the laboratory efficacy of naked *versus* polymer‐complexed dsRNA in inducing mRNA downregulation lethal effects in *B*. *aeneus* through dietary exposure via treated anthers. Based on preliminary screening, a single synthetic cationic polymer was selected as the nanocarrier. The primary objective was not to identify the most effective delivery system, but rather to determine whether complexation with a synthetic polymer could enhance dsRNA stability and its biological activity. The chosen polymer served as a well‐defined and reproducible model system, enabling a focused assessment of the specific contribution of dsRNA complexation to protection against degradation. This foundational approach establishes a basis for future studies aimed at optimizing nanocarrier selection to improve RNAi efficacy in *B. aeneus*.

## 2 MATERIALS AND METHODS

### 2.1 Insects and plant material

Adult *B. aeneus* were collected from untreated (pesticide free) oilseed rape fields near Merchtem, Belgium (50°56′54.0” N 4°12′53.1″ E) in 2023 (for the dsRNA degradation experiment), and near Tartu, Estonia (58.364715° N, 26.666138° E) in 2023 (for the feeding experiment). Pollen beetles were identified using Kirk‐Spriggs,^59^ and only the species *B. aeneus* was used in the experiments. Insects were kept in a growth chamber (MLR‐351H; Sanyo, Osaka, Japan) at 20 °C and 70% relative humidity (RH), with a 16 h:8 h, light: dark photoperiod, and fed *ad libitum* on pollen of from excised flowers of oilseed rape.

Oilseed rape plants used for anther collection for the feeding study were obtained from pesticide free fields in Õssu, Tartu County, Estonia (58.36377° N, 26.66145° E). Plants were taken from the field at an early growth stage (stem elongation stage, BBCH 30–35^60^), and planted into 3‐L pots with N‐P‐K‐fertilized and limed soil (pH 6). They were maintained in a 3 × 3 m climate chamber (Flohr Instruments, Utrecht, Netherlands) at 6 °C and 60 ± 5% RH with a 12 h:12 h, light: dark photoperiod. Before the start of the experiment, the temperature was increased to 20 °C with a 16 h:8 h, light: dark photoperiod, other variables remaining constant. When the oilseed rape plants reached the flowering stage (BBCH 61–69), anthers were collected from blooming flowers using tweezers.

### 2.2 Double‐stranded RNAs

The dsRNA sequences used in the experiments were selected from the existing pollen beetle RNAi literature.^51^, ^52^, ^53^, ^56^, ^57^, ^58^ For the feeding experiment, a 455‐bp dsRNA sequence complementary to an mRNA region of *green fluorescent protein* (dsGFP) was used as a control. For the dsRNA targeting *B. aeneus coatomer subunit α* (dsαCOP), a 213‐bp region of the coding sequence was selected. Likewise, a 454‐bp region was selected for the dsRNA targeting *B. aeneus*'s homolog of the SPT16 protein (dsdre4). The dsRNA fragments were synthesized *in vitro* by AgroRNA (Genolution, Seoul, South Korea) and shipped in distilled water (dH_2_O) at ambient temperature, and kept at 5 ± 1 °C once received. The presence of dsGFP, dsαCOP and dsdre4 stocks was confirmed via gel electrophoresis, indicating absence of nucleic contaminants. To confirm the release of dsRNA from the polymer nanocarrier, mRNA downregulation was assessed using a 495‐bp dsMan2b (*mannosidase 2B*) fragment.

### 2.3 Determination of gut pH

Gut pH was determined using pH indicators 0.1% thymol blue and 0.1% phenol red, each mixed with sugar water solution (final sugar concentration 25%), and administered orally. Insects were starved for 24 h before the experiment. After dissection of the gut, pH was determined by the color of the indicator. A total of 25 adult insect guts were examined with each pH indicator. This method has previously been used for gut pH estimation in insects and mites.^61^, ^62^

### 2.4 Polymer synthesis and characterization

Poly(2‐aminoethyl methacrylate) (PAEMA) and poly(2‐guanidinoethyl methacrylate) (PGUMA) were synthesized using previously described protocols.^63^, ^64^ Briefly, the AEMA monomer was dissolved in acidified double‐distilled water (pH 4, adjusted with HCl) to a concentration of 0.5 m. The free radical polymerization was performed using ammonium persulfate (APS) as an initiator and monomer‐to‐initiator ratios of 25:1 and 50:1 were used. The solutions were degassed using argon gas for 30 min, after which the reaction mixture was heated to 70 °C to start the polymerization. The reaction was continued for 24 h, after which the polymer was purified using dialysis for 24 h against acidified double‐distilled water (pH 4, adjusted with HCl). Dialysis membranes with a molar mass cut‐off of 3.5 kg mol^−1^ (SpectraPor) were used and after dialysis, the polymer solution was lyophilized to obtain the final product.

Guanidinylation of the primary amines of PAEMA was performed by dissolving 300 mg (1.88 mmol of amine functional groups) of the polymer in 4.5 mL double‐distilled water, followed by the addition of 1‐H‐pyrazole‐1‐carboxamidine hydrochloride (HPC) and triethylamine (TEA). We used 1.1 equivalents of TEA (2.07 mmol, 290 μL), as well as 0.5 (0.94 mmol, 138 mg) or 1.0 (1.88 mmol, 276 mg) equivalents of HPC, depending on the intended degree of guanidinylation (50% and 100%, respectively) (Table 1). The reaction was stirred at room temperature (RT) for 24 h, after which the polymer was again purified using dialysis for 24 h against acidified double‐distilled water (pH 4, adjusted with HCl) using dialysis membranes with a molar mass cut‐off of 3.5 kg mol^−1^ (SpectraPor). The final polymer was then obtained by lyophilization.

**Table 1:** Outline of the synthesized polymers investigated in this study

Polymer	Degree of guanidinylation (%)	Number‐average molar mass (kg mol^−1^)	Polydispersity index
PAEMA LMM	/	42.0^	3.8
PGUMA50 LMM	53	47.6*	
PGUMA100 LMM	93	52.0*	
PAEMA HMM	/	64.5^	3.9
PGUMA50 HMM	52	81.9*	
PGUMA100 HMM	92	89.8*	

* The molar mass of poly(2‐guanidinoethyl methacrylate) (PGUMA) was calculated based on the molar mass of the respective PAEMA as obtained through size exclusion chromatography (SEC) (^), taking into account the mass increase resulting from guanidinylation (and assuming that the degree of polymerization remains unchanged).

HMM, high molar mass; LMM, low molar mass.

The synthesized polymers were analyzed using proton nuclear magnetic resonance (^1^H NMR) spectroscopy and Fourier transform infrared (FTIR) spectroscopy to determine their chemical structure, and size exclusion chromatography (SEC) for their molar mass. For the ^1^H NMR measurements, the polymer was dissolved in deuterium oxide [99.9% + 0.1% 3‐(trimethylsilyl)propionic‐2,2,3,3‐d4 acid sodium salt (TMSP)] and the spectra were obtained using an Avance II Ultrashield 400 MHz NMR spectrometer (Bruker, Kontich, Belgium). The spectra were analyzed using the mestrenova software (v14.2.1–27 684). The degree of guanidinylation was determined by taking the ratio of the integration of the peak at 3.6 ppm (i.e. corresponding to the methylene protons next to the guanidine group) and the sum of the peaks at 3.6 and 3.38 ppm (i.e. corresponding to the methylene protons next to the guanidine and amine groups, respectively). FTIR spectra were recorded by a Frontier FT‐IR (PerkinElmer, Leuven, Belgium) and processed using prism (v8.02; GraphPad, San Diego, CA, USA). The SEC setup consisted of a Waters Alliance e2695 separation module, two Shodex OHpak SB‐806 M HQ columns and a Waters 2414 differential refractive index detector (Waters, Antwerpen, Belgium). The mobile phase consisted of an aqueous buffer with 4% (w/v) of sodium dihydrogen phosphate and 3% (w/v) acetonitrile. The polymer and the monodisperse dextran standards (for calibration) were dissolved in this buffer at a concentration of ≈10 mg mL^−1^. The obtained data were analyzed using empower2 software (v3.8.0). The chemical structure and composition of synthesized polymers were confirmed by 1H NMR and FTIR spectroscopy (Supporting Information, Fig. S1–S4). Although FTIR allows the introduction of the C=N stretch of the guanidine group around 1645 cm‐^1^ to be followed, the degree of guanidinylation was determined using 1H NMR spectroscopy by taking the ratio of the methylene peaks next to the oxygen (O) in PAEMA and PGUMA.

### 2.5 Polyplex formation, characteristics and dsRNA degradation assay

Polyplexes were prepared by mixing the required amounts of a 1000 ng μL^−1^ polymer stock solution and the dsRNA stock solutions. The required amount of dsRNA solution was diluted with Milli‐Q water to obtain a final concentration of 20 ng μL^−1^. Although this leads to differences in molar concentrations owing to varying dsRNA lengths, using a uniform mass‐based concentration allows for consistent preparation and facilitates direct comparison of biological effects across treatments. The required amount of polymer was determined based on the nitrogen; phosphorus (N/P) ratio (which varied between 0.8 to 4.2), the mass per charge of the dsRNA (325 g mol^−1^) and the mass per charge of the polymer (calculated based on the degree of guanidinylation, the molar mass of 165.62 g mol^−1^ for the PAEMA repeating unit, and the molar mass of 203.66 g mol^−1^ for the PGUMA repeating unit). The polyplex solution was carefully mixed by inverting the tubes a few times.

After complexation, polyplex solutions were incubated for 30 min at RT (22 ± 1 °C) before loading on a 1.5% agarose gel in 0.5x TAE buffer (Tris‐acetate‐EDTA). After electrophoresis (100 V for 30 min in 0.5x TAE buffer), gels were stained in an aqueous ethidium bromide solution (0.5 mg L^−1^) for 15 min at RT and visualized using a GelDoc XR+ system with image lab software (BioRad, Hercules, CA, USA). The presence of a band at the expected size of the dsRNA indicates the presence of noncomplexed dsRNA. The absence of a band at the expected size of the dsRNA indicates complete polymer–dsRNA complexation. Polyplexes size, polydispersity index (PDI) and zeta potential were measured with dynamic light scattering (DLS) using with a Zetasizer Nano‐ZS (Malvern Panalytical, Malvern, UK) device equipped with a 4 mW He—Ne laser at 633 nm. The polyplex solutions were transferred to disposable cuvettes for the measurements of the polyplex size and PDI, after which they were again transferred to a disposable folded capillary cell for the zeta potential measurements.

The stability of the polyplexes was assessed in three different buffered solutions: phosphate‐buffered saline (PBS), acetic‐and citric acid. To mimic the physiological conditions of the adult *B. aeneus* gut, all solutions were adjusted to pH 5–6 using HCl or NaOH. Two PAEMA polymers (PAEMA LMM and PAEMA HMM) and four PGUMA polymers (PGUMA50 LMM, PGUMA100 LMM, PGUMA50 HMM and PGUMA100 HMM) were screened for their ability to protect the dsRNA against degradation by nucleases in the gut fluid of adult pollen beetles. Gut fluid extracts were prepared by dissecting and collecting four guts in 100 μL PBS solution (adjusted with HCl, to the same pH as estimated in the guts). After centrifugation, 35 μL gut extract was added to 50 μL polyplex solution containing 1000 ng dsRNA, with an N/P ratio of 2.2 using the polymer nanocarrier. The N/P ratio was selected based on DLS measurements. The polyplexes were incubated in the gut extract for different lengths of time: 0, 15, 30 and 60 min. Subsequently, extended periods of 4 h and 24 h also were used to assess the stability of the polyplexes. As a control, naked dsRNA was incubated with the gut extract. To visualize the stability of the dsRNA, the polyplexes were decomplexed by adding 30 μL of a 1% sodium dodecyl sulfate (SDS) solution after initial incubation, followed by 15 min at RT before loading onto the gel. The SDS not only decomplexes the polyplex, but also inhibits the enzymatic reaction of the nucleases.

### 2.6 dsRNA release from polymer nanocarrier confirmed via qPCR

An experiment was carried out to verify the release of dsRNA from the nanocarrier in an assay using *Leptinotarsa decemlineata* Lepd‐SL1 cells. A total of 100 ng dsRNA (dsMan2b), naked or complexed (N/P ratios: 1.2, 2.2 and 4.4), was added to 100 000 Lepd‐SL1 cells. After 48 h of incubation, the cells were washed and RNA was extracted using the RNeasy Mini Kit (Qiagen, Hilden, Germany). RNA concentration was assessed using a Nanodrop spectrophotometer (Thermo Fisher Scientific, Wilmington, NC, USA). RNA purity was verified via gel electrophoresis. Residual genomic DNA was removed using a Turbo DNA‐Free Kit (Invitrogen, Carlsbad, CA, USA), following manufacturer's instructions. The cDNA synthesis was performed using 1 μg of total RNA, using the SuperScript IV cDNA Synthesis kit (Invitrogen), following manufacturer's instructions. Downregulation efficacy was determined by quantitative (q)PCR, as described below.

### 2.7 Feeding assay

In the feeding experiment, oilseed rape anthers were dipped for 15 s in various treatments. Treatments containing PAEMA LMM were used with an N/P ratio of 2.2. A total of 11 different treatments were used: Milli‐Q water; PAEMA LMM; dsGFP (600 ng μL^−1^); dsαCOP (60 ng μL^−1^); dsdre4 (60 ng μL^−1^); dsαCOP (600 ng μL^−1^); dsdre4 (600 ng μL^−1^); dsdre4 (60 ng μL^−1^) + PAEMA LMM; dsαCOP (60 ng μL^−1^) + PAEMA LMM; dsdre4 (600 ng μL^−1^) + PAEMA LMM; dsαCOP (600 ng μL^−1^) + PAEMA LMM. To lower the surface tension, 300 ppm trisiloxane alkoxylate (Silwet 806) was added to each treatment solution. Freshly treated anthers with treatments (dsRNA complexed with nanocarrier and without) were provided daily until the end of the experiment (12 days). After treatment, the anthers were left to air‐dry and then placed in insect rearing cages (transparent, polystyrene, ventilated insect breeding dishes, diameter 10 cm, height 4 cm; SPL Life Sciences, Gyeonggi‐do, South Korea), hereinafter referred to as ‘cages’, along with 10 randomly selected active beetles. The insects were starved for 24 h before the experiments. Survival of the insects was checked daily for ≤12 days, and deceased insects were removed from the cages daily. After the Day (D)1, any deceased beetles were removed from the experiment, as this early stage mortality was likely owing to stress from handling and environmental changes rather than RNAi effects. These initial mortalities (on D1) were minimal (<0.1%) and were not included in the statistical analysis. Each treatment consisted of five replications, with ten insects in each cage, and was repeated three times (*n* = 15; total of 150 insects per treatment).

Relative mRNA expression analysis for all treatments was conducted using qPCR at the time points of D3 and D6 after initial dietary exposure. Two insects were removed from each cage on D3 and D6after the start of each experimental replicate, leaving six insects per cage, respectively, for survival analysis. The removal of insects for qPCR was accounted for in the statistical analysis of survival.

The maintenance of the insects in both feeding experiments was carried out under the same conditions as described above (section 2.1), ensuring consistency with the previous experiment.

### 2.8 qPCR analyses

Insects collected for qPCR were homogenized in 600 μL f RLT (RNA Lysis Tissue buffer; with 10 μL β‐mercaptoethanol added per 100 μL), using sterilized plastic pestles and Eppendorf tubes; the samples were stored at −80 °C until further analysis. Total RNA was extracted using the RNeasy Mini Kit (Qiagen, Venlo, The Netherlands). RNA concentration and purity were measured using a Nanodrop spectrophotometer (Thermo Fisher Scientific), and further assessed by gel electrophoresis. Genomic DNA was removed using the Turbo DNA‐Free Kit (Invitrogen) according to the manufacturer's instructions. The cDNA was synthesized from 1 μg total RNA using the FIREScript RT cDNA Synthesis Kit (Solis BioDyne, Tartu, Estonia). qPCR was performed using the QuantStudio 5 Real‐Time PCR System (Applied Biosystems/Thermo Fisher Scientific). The reaction mixture contained 4 μL of 5 × HOT FIREPol EvaGreen qPCR Supermix (Solis BioDyne, Tartu, Estonia), 0.5 μL of 10 μm (0.25 μm) forward primer (Microsynth, Balgach, Switzerland; Table S1), 0.5 μL of 10 μm (0.25 μm) reverse primer (Microsynth; Table S1), 14 μL PCR‐grade water and 500 ng cDNA, bringing the total volume to 20 μL.

Amplification conditions were as follows: 15 min at 95 °C, 37 cycles of 15 s at 95 °C and 1 min at 59 °C, followed by a melting‐curve analysis with a temperature range of 60–95 °C. The reactions were set up in 96‐well PCR plates in triplicate. Normalization of data was performed using the two housekeeping genes *rps3* and *act*. Relative mRNA expression values were calculated using qBase+, which incorporates multiple reference gene normalization and statistical error estimation, extending beyond the classical 2^−ΔΔCt^ method. Nontemplate and no‐reverse transcriptase controls were included, and reference gene stability was evaluated within the software.

### 2.9 Statistical analysis

Regarding all mortality analyses, comparisons were made between dsGFP and all concentrations of both naked and complexed dsαCOP and dsdre4. For mortality assessment, we adjusted the death rate in the treatment groups relative to the control group, which received the food source without dsRNA (Milli‐Q water). For survival analysis, the homogeneity of variance and normality of data distributions were determined using the Levene and Shapiro–Wilk tests, respectively. Because the data distribution did not follow a normal distribution, the Kruskal–Wallis test was used as a nonparametric alternative to ANOVA; this was followed by the Wilcoxon rank‐sums test, with Bonferroni correction, for *post hoc* pairwise comparisons.

For mRNA expression analyses, comparisons were made between all dsRNA concentrations of naked and complexed dsαCOP and dsdre4. Gene expression comparisons were statistically evaluated using one‐way ANOVA (because the data follow normal distribution) to assess the effects of targeting the *dre4* and *αCOP* genes.

All statistical analyses of mortality were done in R v3.6.3 (R Foundation for Statistical Computing, Vienna, Austria), using R packages tidyverse ^65^, tidyr,^66^ dplyr,^67^ knitr,^68^ broom,^69^ statix,^70^ emmeans,^71^ rmisc,^72^ multcomp,^73^ readxl,^74^ ggplot2 ^74^ and forcats.^75^

## 3 RESULTS

### 3.1 Assessment of dsRNA–Polymer complex stability and efficacy in simulated and cellular environments

In order to evaluate the stability and efficacy of polymer‐complexed dsRNA, a stepwise approach was undertaken. As a first step, the gut pH of *B. aeneus* was determined. This was done by orally administering pH indicators, 0.1% thymol blue and 0.1% phenol red, dissolved in a sugar water solution (final sugar concentration of 25%). The analysis revealed a mildly acidic midgut environment, with a pH ranging from 5 to 6, and no evidence of an alkaline foregut (Fig. S5).

Following the determination of gut pH, the next step was to evaluate the stability and protective capacity of polymer‐complexed dsRNA under physiologically relevant conditions. We determined the optimal N/P ratio using both gel electrophoresis and DLS. Complete complexation was observed starting at N/P ratio 1.6 in gel electrophoresis (Fig. S6) and 1.8 in DLS (Fig. S7). The optimal N/P ratio was 2.2, as this resulted in polyplexes with the smallest particle sizes (89.48 nm), acceptable PDI (0.318) (Table S2), and a stable positive zeta potential. Lower ratios resulted in oversized particles and unstable charge, whereas higher polymer amounts could increase nonspecific toxicity. Thus, N/P 2.2 offers the best balance between efficiency, stability and safety.

The stability of both naked dsRNA and the different dsRNA–polymer polyplexes was assessed at the previously determined gut pH using three different buffers, all adjusted to the gut pH. This analysis showed that all six polyplex formulations remained stable under acidic conditions, warranting their further evaluation in protecting dsRNA from enzymatic degradation in the gut of *B. aeneus*. To this end, dsGFP was complexed with four PGUMA polymers (PGUMA50 LMM, PGUMA100 LMM, PGUMA50 HMM, and PGUMA100 HMM) and two PAEMA polymers (PAEMA LMM and PAEMA HMM) at an N/P ratio of 2.2, and incubated with *B. aeneus* gut fluid extracts. To confirm complex formation and assess dsRNA integrity, the samples were treated with SDS to dissociate the polyplexes and release any intact dsRNA. The presence of detectable dsRNA after 24 h of incubation and subsequent SDS treatment confirmed that the polymers successfully formed stable complexes, protecting the dsRNA from degradation (Fig. 1). Among the tested formulations, PAEMA LMM was selected for further experiments. This choice was primarily based on its superior performance in the stability assay, where it consistently formed protective polyplexes with dsRNA over 24 h. Notably, electrophoretic analysis revealed a shift in the band of the naked dsRNA incubated with gut extract, suggesting partial degradation or fragmentation. By contrast, dsRNA complexed with PAEMA LMM retained its original band size, indicating protection against nuclease activity. Additionally, narrowing the study to a single nanocarrier was necessary to simplify downstream experiments and focus on the most promising candidate.

**Figure 1:**
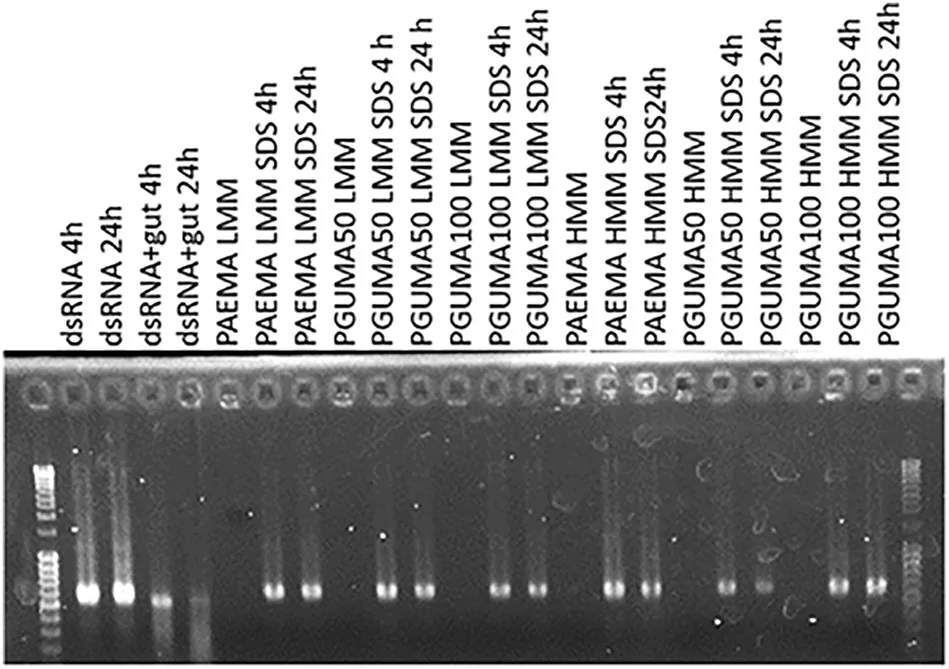
Effect of polymer chemical structure and molar mass on the dsRNA stability in *B. aeneus* gut as analyzed by an electrophoretic mobility assay. Polyplexes were prepared between dsGFP and two poly(2‐aminoethyl methacrylate) (PAEMA) polymers (PAEMA LMM and PAEMA HMM) or four poly(2‐guanidinoethyl methacrylate) (PGUMA) polymers (PGUMA50 LMM, PGUMA100 LMM, PGUMA50 HMM and PGUMA100 HMM) at an N/P ratio of 2.2 and subsequently incubated in *B. aeneus* gut extract for 4 h and 24 h. LMM, low molar mass; HMM, high molar mass.

In order to further evaluate the functionality of the selected polyplexes, a cell‐based assay was conducted using *L. decemlineata* Lepd‐SL1 cells. This experiment aimed to validate the ability of the polymer nanocarrier (PAEMA LMM) to release its dsRNA cargo in the intra cellular environment. The qPCR analysis demonstrated a significant reduction in target gene expression. Compared to the untreated control, naked dsRNA induced a 2.52‐fold decrease (*P* > 0.05), whereas treatment with PAEMA LMM at N/P 2.2 further reduced expression to 0.353‐fold relative to naked dsRNA (*P* > 0.05). These results indicate that the dsRNA was not only released from the polyplex, but also remained functional, entered the cells, and effectively triggered RNAi interference, thus both releasing from the polyplex and remaining functional (Fig. S8). By contrast, treatments with PAEMA LMM at N/P ratios of 1.2 and 4.4 resulted in increased target gene expression compared to naked dsRNA, with fold changes of 0.923 and 0.225, respectively (*P* > 0.05), suggesting reduced RNAi efficacy or suboptimal delivery at these ratios.

### 3.2 *Brassicogethes aeneus* mortality and mRNA expression – feeding assay on anthers treated with nanocomplexed dsRNA and naked dsRNA

Despite the enhanced stability of polyplexed dsRNA and its confirmed ability to induce target gene silencing, feeding on anthers treated with polyplexed dsRNA did not lead to a significant reduction in pollen beetle survival. By contrast, by the end of the trial on D12, mortality in the group exposed to anthers treated with naked dsαCOP at 600 ng μL^−1^ had increased to 53%, up from 32% on D7 (F = 12.43, *P* < 0.01; Fig. 2). No statistically significant difference in survival was observed in beetles fed anthers treated with dsdre4, whether polyplexed or naked, compared to the control group.

**Figure 2:**
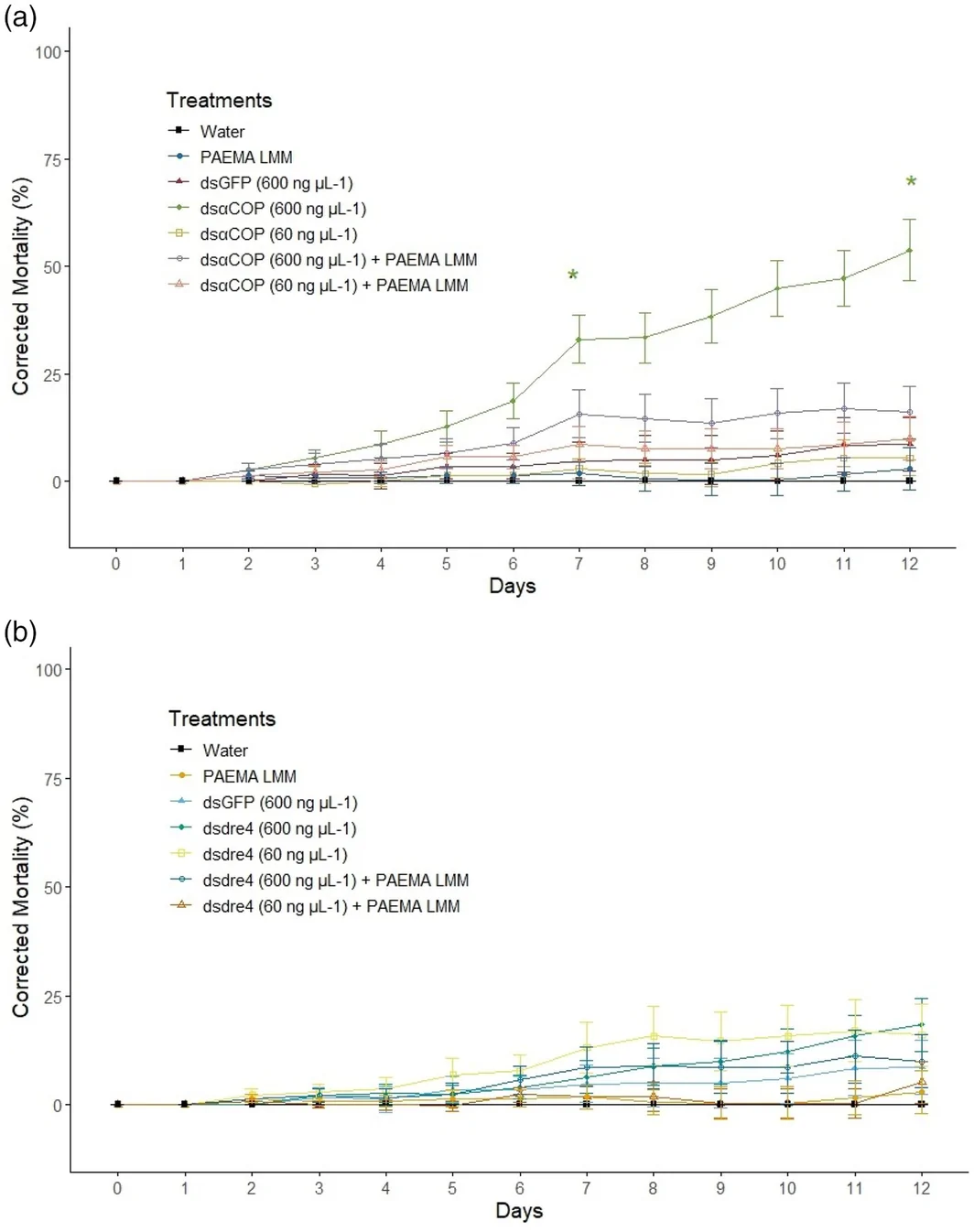
Corrected mortality (%) of *B. aeneus* adults fed with (a) dsαCOP (60 ng μL^−1^, 600 ng μL^−1^) and (b) dsdre4 (60 ng μL^−1^, 600 ng μL^−1^), each with and without the polymer poly(2‐aminoethyl methacrylate) of low molar mass (PAEMA LMM), across three experimental replicates. Mortality values were corrected using the water control (Milli‐Q water). Asterisks (*) denote statistically significant differences between dsαCOP or dsdre4 treatments and the dsGFP control (*P* < 0.05, Kruskal–Wallis test followed by Wilcoxon rank‐sum test with Bonferroni correction). The color of the asterisk corresponds to the respective treatment group. Error bars represent ± SEM.

Although dietary exposure to anthers treated with dsαCOP‐PAEMA LMM polyplexes did not result in significantly increased mortality compared to the control, a reduction in αCOP transcript levels was observed (Fig. 3). Six days after the initial exposure, beetles fed dsαCOP–PAEMA LMM at 60 ng μL^−1^ exhibited a 0.77‐fold reduction in *αCOP* transcript levels relative to naked at dsαCOP at 600 ng μL^−1^ (t=1.77, df=3.0, *P* > 0.05). Overall, insects fed with anthers treated with dsαCOP at 60 ng μL^−1^ displayed lower *αCOP* transcript levels compared to those treated with dsGFP (control). On D6, a significant reduction in *αCOP* expression was observed, with a 0.53‐fold change compared to the dsGFP control (t= 1.75, df=2.1, *P* < 0.05). By contrast, no reduction in *dre4* transcript levels was detected following treatment with either naked dsdre4 or dsdre4–PAEMA LMM polyplexes. Interestingly, instead of downregulation, a trend toward upregulation of *dre4* expression was observed in some treatment groups. Specifically, elevated transcript levels were noted on D3 and D6 in the group treated with 60 ng μL^−1^ dsdre4–PAEMA LMM polyplexes compared to the naked dsdre4 control. However, these differences were not statistically significant (*P* > 0.05).

**Figure 3:**
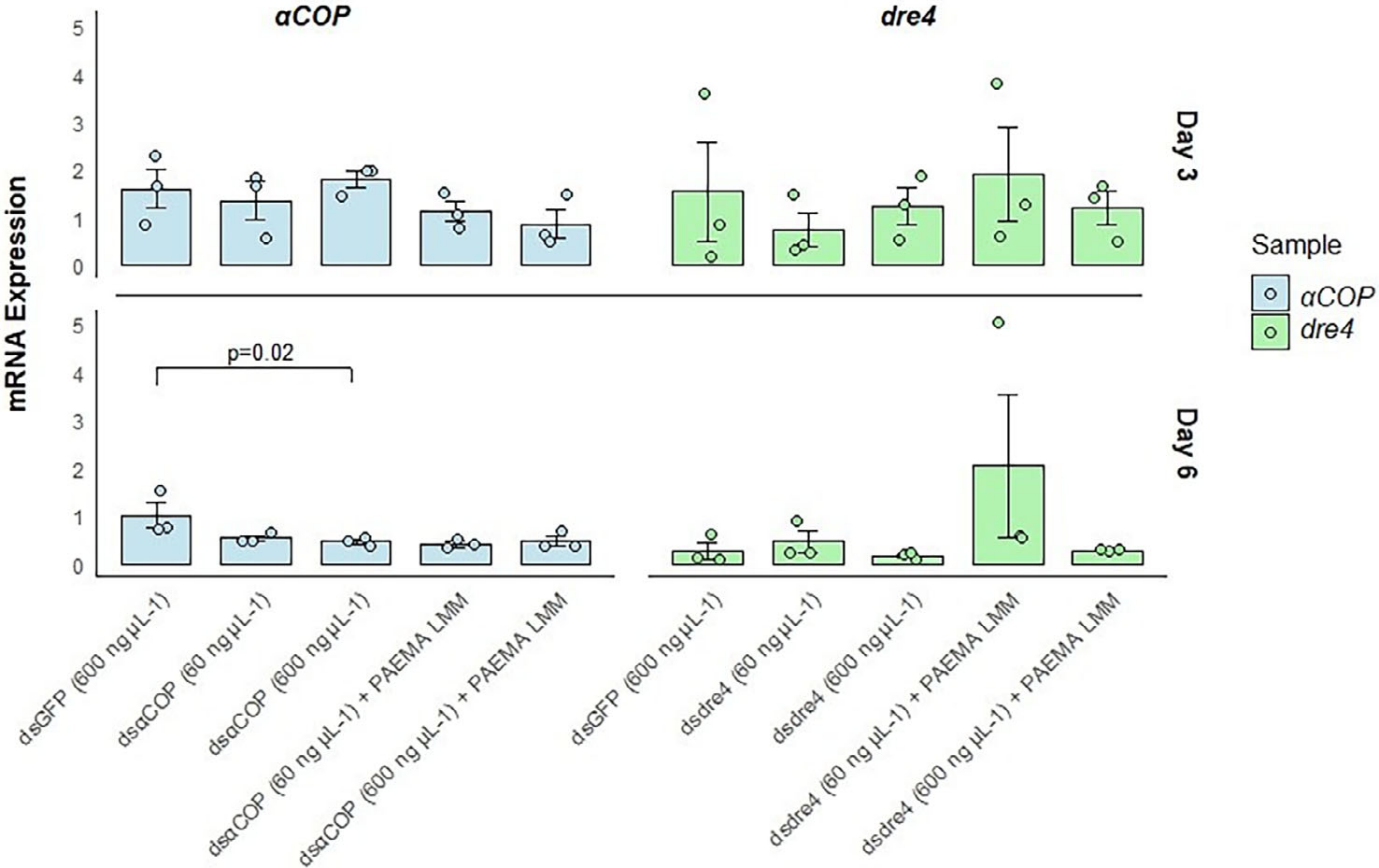
Relative mRNA expression of complexed dsRNA (with poly(2‐aminoethyl methacrylate) of low molar mass, PAEMA LMM) in *B. aeneus* was measured at D3 and D6 after initial dietary exposure to treatments (*n* = 3 biologically independent qPCR samples per treatment and time point). Relative GFP mRNA expression was assessed using qBase without manual setting of calibration groups. Normalization was performed using the reference genes *rps3* and *act*, and relative mRNA values were calculated automatically by qBase. Therefore, control group values are shown as computed by qBase and may differ from 1.0. Error bars represent the SEM from biological replicates. *P*‐values above brackets indicate significance of dsαCOP treatment compared to dsGFP controls (*P* < 0.05; one‐way ANOVA). All data are presented on the same scale for comparison.

## 4 DISCUSSION

This study evaluated whether selected polymeric nanocarriers could enhance RNAi efficacy in *B. aeneus* by delivering dsRNA targeting two genes, *dre4* and *αCOP*, via dietary exposure through anther feeding. Although *αCOP* had previously demonstrated potential as a lethal target, this marks the first evaluation of *dre4* using this delivery method.

PAEMA LMM provided a stable nanoparticle formulation and enhanced knockdown of *αCOP* transcript levels by D6, yet this did not translate into increased mortality. In fact, mortality was significantly higher (50%) in beetles fed with naked dsαCOP compared to just 15% with PAEMA LMM–dsαCOP polyplexes. These findings suggest that while complexation may enhance dsRNA stability and cellular uptake, persistence of dsRNA alone does not guarantee RNAi‐induced lethality. The discrepancy may stem from downstream physiological responses or compensatory mechanisms triggered by the dsαCOP‐PAEMA LMM polyplex, which could mitigate lethal outcomes despite transcript silencing. Previous studies have shown that RNAi‐induced mortality is highly context‐dependent. For example, in *B. aeneus*, oral delivery of naked dsαCOP led to mortality rates in the range 43–89%, with stronger effects at higher concentrations and more pronounced transcript suppression.^53^ However, other insect studies have reported substantial knockdown without corresponding mortality, particularly in species with lower RNAi sensitivity or robust compensatory mechanisms.^76^ A recent study on *L. decemlineata* demonstrated that chitosan‐based dsRNA formulations significantly improved dsRNA stability, yet did not enhance insecticidal efficacy, further underscoring the complexity of RNAi outcomes *in vivo*.^77^ Furthermore, RNAi can induce sublethal effects such as developmental delays, reduced fertility or behavioral changes, which may not be immediately evident in mortality assays. These effects are increasingly recognized as ecologically relevant endpoints in RNAi‐based pest management.^78^, ^79^, ^80^ The discrepancy between transcript silencing and phenotypic outcomes may stem from differences in gene function, tissue‐specific expression, or the physiological impact of delivery formulations. In this study, such sublethal effects were not assessed but could provide further insight into the biological consequences of αCOP knockdown.

Likewise, neither naked nor complexed dsdre4 led to a significant reduction in beetle survival or gene expression. A slight nonsignificant upregulation of *dre4* was observed on D3 and D6 in beetles fed 60 ng μL^−1^ of complexed dsdre4. Similar trends have been reported in other insects and may reflect stress responses or feedback mechanisms rather than genuine gene activation.^81^, ^82^, ^83^ These findings highlight the need for time‐course studies with appropriate controls to better capture RNAi dynamics. Moreover, the low dsRNA concentration, limited by the polymer's solubility, may have further restricted RNAi efficacy for this target.

Comparative studies in other coleopteran species have shown that improved delivery via nanoparticles does not always lead to insect mortality.^84^ In some cases, gene knockdown occurs without a corresponding phenotypic effect, which is likely to result from the nonessential nature of the target gene or insufficient systemic spread. Nonetheless, PAEMA LMM maintained dsRNA integrity and facilitated transcript knockdown, confirming its potential as a viable nanocarrier. Future research should integrate advanced imaging and transcriptomic tools to assess dsRNA uptake, siRNA production, and potential off‐target or compensatory responses to fully elucidate the underlying limitations.

## 5 CONCLUSION

This study demonstrates that although PAEMA LMM enhances dsRNA stability and facilitates target gene knockdown in *B. aeneus*, it does not necessarily improve RNAi‐induced mortality. The highest mortality (~50%) was achieved with uncomplexed dsαCOP, while complexed forms resulted in lower lethality despite transcript silencing. For *dre4*, neither delivery approach was effective in inducing gene silencing or mortality.

These results highlight the complex interplay between dsRNA delivery, gene targeting and physiological responses in RNAi‐based pest control. They underscore the need for carefully tailored delivery strategies and further mechanistic insights to optimize the use of nanocarriers like PAEMA LMM. Future work should prioritize identifying gene targets with stronger lethal phenotypes, improving carrier design for controlled release, and validating uptake and downstream effects using integrated molecular and imaging approaches. Together, these advances will be critical to harness the full potential of RNAi for sustainable pest management in *B. aeneus* and related species.

## CONFLICT OF INTEREST

The authors declare that they have no known competing financial interests or personal relationships that could have appeared to influence the work reported in this paper.

## AUTHOR CONTRIBUTIONS

TK, SilvaV, RK, CNTT, PD, LVD, SvenV, JW, KDS and EV designed research. TK, LS, IC, SilvaV, RK performed the experiments. TK, CNTT, JW, KDS, EV, PD participated in the interpretation of the results. LVD and SvenV synthesized and performed characterization of the polymer nanocarrier and the physicochemical evaluation of the polyplexes. TK and AIS performed data analysis and visualization. TK, JW, KDS, CNTT and EV wrote the first draft of the manuscript. All authors contributed to revision and edited the manuscript. All authors read and approved the manuscript.

## Supporting information


**Figure S1.** Superimposed ^1^H NMR spectra of the three low molar mass: one poly(2‐aminoethyl methacrylate) [PAEMA LMM (red)] and two poly(2‐guanidinoethyl methacrylate) (PGUMA) polymers [PGUMA50 LMM (green); PGUMA100 LMM (blue)].
**Fig. S2.** Superimposed FTIR spectra of the three low molar mass polymers used in this work: one poly(2‐aminoethyl methacrylate) [PAEMA LMM (red)] and two poly(2‐guanidinoethyl methacrylate) (PGUMA) polymers [PGUMA50 LMM (green); PGUMA100 LMM (blue)] with the stretches and bands.
**Fig. S3.** Superimposed ^1^H NMR spectra of the three high molar mass polymers used in this work: one poly(2‐aminoethyl methacrylate) [PAEMA HMM (red) and two poly(2‐guanidinoethyl methacrylate) PGUMA50 HMM (green); PGUMA100 HMM (blue)].
**Fig. S4.** Superimposed FTIR spectra of the three high molar mass polymers used in this work: one poly(2‐aminoethyl methacrylate) [PAEMA LMM (red) and two poly(2‐guanidinoethyl methacrylate) PGUMA50 LMM (green); PGUMA100 LMM (blue)] with the stretches and bands.
**Fig. S5.** *Brassicogethes aeneus* gut pH level determined using pH indicators, 0.1% thymol blue and 0.1% phenol red. (a) pH comparison chart, and (b) gut after consuming food with pH indicator.
**Fig. S6.** Determining the N/P ratio at which the dsRNA was fully complexed: polyplexes were prepared by mixing polymers (in this case PAEMA LMM) with dsRNA (dsGFP 200 ng μL^−1^) at varying N/P ratios (1.0–2.2) and incubated at room temperature for 30 min. Samples were analyzed by 1.5% agarose gel electrophoresis stained with ethidium bromide. The presence of a band corresponding to free dsRNA indicates incomplete complexation, whereas absence of the band demonstrates successful polymer‐dsRNA complex formation.
**Fig. S7.** Characterization of PAEMA‐dsRNA polyplexes at different N/P ratios: (a) *z*‐average size, (b) number‐average size and (c) zeta potential of PAEMA‐dsRNA polyplexes at different N/P ratios were measured by dynamic light scattering (DLS). The data represent mean values ± standard deviation.
**Fig. S8.** Ability of low molar mass poly(2‐aminoethyl methacrylate) (PAEMA LMM) to release 100 ng dsRNA from the polyplex at N/P ratios of 1.2, 2.2 and 4.4 after 48 h incubation (*n* = 3 biologically independent qPCR samples per treatment and time point). Control is no treatment. Data were normalized against a calibrator sample (untreated control) using the reference genes ARF and RP18, which were specifically selected for this experiment. Relative mRNA expression values were calculated using qBase. Error bars represent the SEM from the biological replicates.
**Table S1.** Primers used in the present studies feeding part.
**Table S2.** Physicochemical characteristics of polyplexes formed by PAEMA LMM and dsGFP (200 ng μL^−1^): size, polydispersity index (PDI), and zeta potential, measured with a Zetasizer Nano‐ZS (Malvern Panalytical) device equipped with a 4 mW He—Ne laser at 633 nm.

## Data Availability

The data that support the findings of this study are available on request from the corresponding author. The data are not publicly available due to privacy or ethical restrictions.
