# Supplementary material for: Synthetic polymer nanocarrier enhances dsRNA stability but not RNAi efficacy in Brassicogethes aeneus
Source: Pest Manag Sci. 2026 Jan 19;82(11):10615–24. doi: 10.1002/ps.70516 (PMC13665585; doi:10.1002/ps.70516)
Supplement: Supplementary file 1 [file PS-82-10615-s001.docx]

**Supplementary Materials:**

**Synthetic polymer nanocarrier enhances dsRNA stability but not RNAi efficacy in *Brassicogethes aeneus***

Triin Kallavus^1,2^*, Liina Soonvald^1^, Iris Callewaert^2^, Ana Isabel Silva^3^, Ericmar Ávila Dos Santos ^2^, Silva Vilumets^1^, Riina Kaasik^1^, Sven Vereecken^4^, Lenny Van Daele^4^, Peter Dubruel^4^, Jonathan Willow^,5^, Clauvis Nji Tizi Taning^2^, Kristof De Schutter^2^, Eve Veromann^1^

1. Chair of Plant Health, Institute of Agricultural and Environmental Sciences, Estonian University of Life Sciences, Tartu 51006, Estonia
2. Department of Plants and Crops, Faculty of Bioscience Engineering, Ghent University, Ghent, 9000, Belgium
3. Center for Magnetic Resonance Research, Department of Radiology, University of Minnesota, MN 55455, USA
4. Polymer Chemistry and Biomaterials Group, Department of Organic and Macromolecular chemistry, Ghent University, Ghent, 9000 Belgium
5. Pheasants Forever and Quail Forever, St. James, MN 56081, USA

**Figure S1.** Superimposed ^1^H NMR spectra of the three low molar mass: one poly(2-aminoethyl methacrylate) (PAEMA LMM (red)) and two poly(2-guanidinoethyl methacrylate) (PGUMA) polymers: PGUMA50 LMM (green); PGUMA100 LMM (blue)).

PAEMA LMM (ppm): 0.90+1.08 (CH3) - 2.01 (-CH2-, backbone) - 3.34 (-CH2-, next to amine) - 4.23 (-CH2-, next to O)

PGUMA50 LMM (ppm): 0.91+1.08 (CH3) - 2.01 (-CH2-, backbone) - 3.38 (-CH2-, next to amine) - 3.60 (-CH2-, next to guanidine) - 4.13 (-CH2-, next to O modified units) - 4.23 (-CH2-, next to O unmodified units)

PGUMA100 LMM (ppm): 0.90+1.06 (CH3) - 2.01 (-CH2-, backbone) - 3.34 (-CH2-, next to amine) - 3.59 (-CH2-, next to guanidine) - 4.13 (-CH2-, next to O modified units) - 4.23 (-CH2-, next to O unmodified units)

**Figure S2.** Superimposed FT-IR spectra of the three low molar mass polymers used in this work: one poly(2-aminoethyl methacrylate) (PAEMA LMM (red)) and two poly(2-guanidinoethyl methacrylate) (PGUMA) polymers: PGUMA50 LMM (green); PGUMA100 LMM (blue)) with the stretches and bands.

PAEMA LMM (cm-1): 1147 (C-O/C-N stretch) – 1608 (N-H bend) – 1717 (C=O stretch) – 2900 (CH2/CH3 stretch) – 3382 (1° N-H stretch)

PGUMA50 LMM (cm-1): 1150 (C-O/C-N stretch) – 1622 (N-H bend) – 1646 (C=N stretch) – 1716 (C=O stretch) – 2995 (CH2/CH3 stretch) – 3147 (2° N-H stretch) – 3333 (1° N-H stretch)

PGUMA100 LMM (cm-1): 1152 (C-O/C-N stretch) – 1619 (N-H bend) – 1644 (C=N stretch) – 1719 (C=O stretch) – 3006 (CH2/CH3 stretch) – 3146 (2° N-H stretch) – 3322 (1° N-H stretch)

**Figure S3.** Superimposed ^1^H NMR spectra of the three high molar mass polymers used in this work: one poly(2-aminoethyl methacrylate) (PAEMA HMM (red) and two poly(2-guanidinoethyl methacrylate) PGUMA50 HMM (green); PGUMA100 HMM (blue)).

PAEMA HMM (ppm): 0.98+1.14 (CH3) - 2.04 (-CH2-, backbone) - 3.36 (-CH2-, next to amine) - 4.29 (-CH2-, next to O)

PGUMA50 HMM (ppm): 0.92+1.09 (CH3) - 2.02 (-CH2-, backbone) - 3.38 (-CH2-, next to amine) - 3.60 (-CH2-, next to guanidine) - 4.13 (-CH2-, next to O modified units) - 4.28 (-CH2-, next to O unmodified units)

PGUMA100 LMM (ppm): 0.92+1.09 (CH3) - 2.02 (-CH2-, backbone) - 3.38 (-CH2-, next to amine) - 3.60 (-CH2-, next to guanidine) - 4.13 (-CH2-, next to O modified units) - 4.28 (-CH2-, next to O unmodified units)

**Figure S4.** Superimposed FT-IR spectra of the three high molar mass polymers used in this work: one poly(2-aminoethyl methacrylate) (PAEMA LMM (red) and two poly(2-guanidinoethyl methacrylate) PGUMA50 LMM (green); PGUMA100 LMM (blue)) with the stretches and bands.

PAEMA HMM (cm-1): 1147 (C-O/C-N stretch) – 1607 (N-H bend) – 1717 (C=O stretch) – 2954 (CH2/CH3 stretch) – 3382 (1° N-H stretch)

PGUMA50 HMM (cm-1): 1148 (C-O/C-N stretch) – 1621 (N-H bend) – 1646 (C=N stretch) – 1717 (C=O stretch) – 2990 (CH2/CH3 stretch) – 3150 (2° N-H stretch) – 3333 (1° N-H stretch)

PGUMA100 HMM (cm-1): 1152 (C-O/C-N stretch) – 1619 (N-H bend) – 1644 (C=N stretch) – 1720 (C=O stretch) – 3002 (CH2/CH3 stretch) – 3146 (2° N-H stretch) – 3319 (1° N-H stretch)

**Figure S5.** *Brassicogethes aeneus* gut pH level determined using pH indicators, 0.1% thymol blue and 0.1% phenol red. (a) pH comparison chart, and (b) gut after consuming food with pH indicator.

**Figure S6.** Determining the N/P ratio at which the dsRNA was fully complexed: **p**olyplexes were prepared by mixing polymers (in this case PAEMA LMM) with dsRNA (dsGFP 200 ng µL-^1^ ) at varying N/P ratios (1.0- 2.2) and incubated at room temperature for 30 minutes. Samples were analyzed by 1.5% agarose gel electrophoresis stained with ethidium bromide. The presence of a band corresponding to free dsRNA indicates incomplete complexation, whereas absence of the band demonstrates successful polymer-dsRNA complex formation.

**Figure S7.** Characterization of PAEMA-dsRNA polyplexes at different N/P ratios: a) z-average size, b) number-average size and c) zeta potential of PAEMA-dsRNA polyplexes at different N/P ratios were measured by dynamic light scattering (DLS). The data represent mean values ± standard deviation.

**Figure S8.** The ability of low molar mass poly(2-aminoethyl methacrylate) (PAEMA LMM) to release 100 ng of dsRNA from the polyplex at N/P ratios of 1.2, 2.2, and 4.4 after 48 hours incubation (n = 3 biologically independent qPCR samples per treatment and time point). Control is no treatment. Data were normalized against a calibrator sample (untreated control) using the reference genes ARF and RP18, which were specifically selected for this experiment. Relative mRNA expression values were calculated using qBase. Error bars represent the standard error of the mean (SEM) from the biological replicates.

**Table S1.** Primers used in the present studies feeding part.

| Gene | Name | Forward (5' to 3') | Reverse (5' to 3') |
| --- | --- | --- | --- |
| *gfp* | green fluorescent protein | CACATGAAGCAGCACGACTT | TGCTCAGGTAGTGGTTGTCG |
| *αCOP* | coatomer subunit alpha | CCAACTTGGTCATTTAACAATCTGG | CCCTTCCTTCATCATGGACA |
| *dre4* | homolog of SPT16 | TGTGGAGCTGCTTCATTTCG | ATATCGCAGGAGTTCAGCCA |
| *rps3* | ribosomal protein S3 | CCAACGCGTACCGAAATTAT | GAGTTTTCGGGGAAGTTGAA |
| *act* | actin | TCACGGACAATTTCCCTTTC | TATCCTCCGTTTGGACTTGG |

**Table S2.** Physicochemical characteristics of polyplexes formed by PAEMA LMM and dsGFP (200 ng µL⁻¹): size, polydispersity index (PDI), and zeta potential, measured with Malvern Zetasizer Nano-ZS (Malvern Panalytical) device equipped with a 4 mW He-Ne laser at 633 nm..

| N/P ratio | Z-average (nm) | SD Z-average (nm) | Number mean (nm) | SD Number mean (nm) | PDI | SD PDI | Zeta potential (mV) | SD Zeta potential (mV) |
| --- | --- | --- | --- | --- | --- | --- | --- | --- |
| 1.0 | 120.50 | 25.24 | 30.80 | 7.81 | 0.33 | 0.05 | -26.20 | 1.69 |
| 1.2 | 97.15 | 2.25 | 43.98 | 18.8 | 0.24 | 0.03 | -27.50 | 2.03 |
| 1.4 | 121.20 | 2.98 | 69.63 | 5.11 | 0.21 | 0.01 | -24.30 | 1.89 |
| 1.6 | 3358.00 | 351.00 | 495.30 | 196.10 | 0.75 | 0.18 | -6.93 | 0.61 |
| 1.8 | 2899.00 | 350.60 | 693.70 | 77.45 | 0.99 | 0.00 | 4.85 | 0.20 |
| 2.0 | 172.20 | 68.26 | 33.54 | 5.57 | 0.36 | 0.04 | 16.30 | 2.70 |
| 2.2 | 89.48 | 3.93 | 26.35 | 11.55 | 0.31 | 0.05 | 24.10 | 0.39 |
| 2.8 | 103.40 | 4.87 | 33.90 | 5.53 | 0.42 | 0.04 | 27.10 | 0.74 |
| 3.4 | 95.57 | 2.51 | 25.34 | 6.79 | 0.42 | 0.03 | 31.10 | 3.74 |
| 4.4 | 98.65 | 4.05 | 27.91 | 8.29 | 0.40 | 0.03 | 23.20 | 1.79 |
